# Editorial: Current trends and challenges in the assessment of suicidal behavior: a psychometric approach

**DOI:** 10.3389/fpsyt.2023.1243062

**Published:** 2023-07-11

**Authors:** Samer El Hayek, Anila Mubashir, S. M. Yasir Arafat

**Affiliations:** ^1^Department of Medical, Erada Center for Treatment and Rehab in Dubai, Dubai, United Arab Emirates; ^2^Department of Applied Psychology, National University of Modern Languages, Rawalpindi, Pakistan; ^3^Department of Psychiatry, Enam Medical College and Hospital, Dhaka, Bangladesh

**Keywords:** suicide, suicidal behavior, psychometric, validation, reliability, scale development

Suicide is a fate of a complex interplay among multiple factors namely genetics, environment, psychology, psychiatric disorders, and social effect (1, 2). In contrast to other public health issues, the death and sufferings due to suicidal behavior have not declined in recent decades. One of the primary obstacles in suicide prevention is the unavailability of reliable measurements. Till to date, precise estimation of risk factors is a daunting task for the clinicians as well as the academicians (3). Psychometric instruments have been developed, and cross-cultural validation of these measurements has been recommended as a way out. This Research Topic aims to accumulate research findings assessing suicidal behaviors in general, with a particular focus on development and validation of psychometric tools measuring suicidal behaviors.

Three studies looked at different suicide-related scales in low- and middle-income countries (LMICs). In one study, Arafat et al. tested the psychometric properties of the *Interpersonal Needs Questionnaire (INQ-15)* and *Acquired Capability for Suicide Scale-Fearlessness About Death (ACSS-FAD)* among 1,207 medical and university students. Both scales revealed acceptable levels of reliability. A two-factor structure of INQ was found by *confirmatory factor analysis* after removing three items from thwarted belongingness domain and a single-factor structure for ACSS-FAD after removing the same number of items. The validation of the INQ and ACSS-FAD enables their utilization in future studies in Bangla, facilitating the exploration of suicide risk factors and the formulation of effective prevention approaches to reduce the risk of suicide among medical college and university students in Bangladesh. van Bentum et al. introduced a new instrument *Suicidal Intrusions Attributes Scale (SINAS)* to measure the severity and pattern of suicidal intrusions and assessed its psychometric properties. The study included 168 individuals with depression and suicidal behavior from outpatient mental health services in the Netherlands. In addition to the 10-item SINAS, authors also used the *Suicidal Ideation Attributes Scale (SIDAS)*, the *Prospective Imagery Task (PIT)*, 4 items of the *Suicidal Cognitions Interview (SCI)*, and the *Beck Depression Inventory (BDI-II)*. The study demonstrated good rliability (Cronbach's α = 0.91) and convergent validity. Only one dimension was extracted by confirmatory factor analysis. The authors concluded that the instrument may be utilized for screening purpose in both research and clinical settings. In their brief report, Rafati et al. performed the validation of the Persian version of the *Predicaments Questionnaire (PQ)*, which measures social attitudes toward suicide, among 151 students. The study revealed acceptable content and face validities of Persian PQ. Confirmatory factor analysis revealed one dimension. Also, it showed high reliability in several parameters (Cronbach's alpha 0.94, McDonald's Omega 0.94, and Intra-class Correlation Coefficient 0.99).

One study by Xu et al. looked at the role of peripheral blood cytokines as potential diagnostic biomarkers of suicidal ideation in major depressive disorder (MDD) patients at their first episode without any history of medication use. The levels of 37 cytokines were compared between 26 patients with MDD without suicidal ideations and 29 patients with MDD and suicidal ideations. Suicidal ideation was determined by the *Beck Scale for Suicide Ideation (BSSI)*. The levels of cytokines CCL26 and VEGF were significantly lower, while IL-17C, CXCL10, and TNF-β levels were significantly higher, in patients with suicidal ideations compared to those without (all *p* < 0.05). While controlling for independent variables including age, sex, body mass index, smoking, and the *Hamilton Depression Rating Scale* 24 scores, group was a significant independent predictor of serum IL-17C, CCL-26, VEGF, and TNF-β levels (all *p* < 0.05). Lastly, a combined panel of IL-17C and TNF-β revealed high accuracy in discriminating those with suicidal ideations compared to controls (Area under the curve = 0.848, sensitivity = 75.9%, specificity = 72.7%), suggesting that circulating IL-17C and TNF-β may bear promise as biomarkers for identification of suicidal ideations in MDD.

In another study, Abdullah et al. examined the adolescent and young adult Pakistani males (747) and concluded that higher religious commitment was associated with lower suicidal ideation, highlighting the potential of religiosity to buffer against suicidal thoughts, particularly among impulsive individuals. Assessment tools employed in the study were BSSI, *Barratt Impulsivity Scale-II (BIS-II), Depression Anxiety Stress Scale (DASS)*, and *Religious Commitment Inventory-10 (RCI-10)*. About one-quarter had a history of suicidal acts (20.2%) and suicidal ideations (23.7%). Impulsivity (predictor) was inversely associated with religious commitment (*r* = −0.33, *p* < 0.01) and religious commitment (mediator) was inversely related to suicidal ideations (outcome) (*r* = −0.32, *p* < 0.01). Furthermore, higher religious commitment reduced the association between impulsivity and suicidal ideations (*p* < 0.01), highlighting the potential of religiosity to buffer against higher suicidal ideation, particularly among impulsive young persons.

Ma et al. assessed suicidal intent and related factors among 225 Chinese patients admitted to the hospital emergency with deliberate acute pesticide poisoning (APP). Patients were investigated using *Beck Suicidal Intent Scale (SIS), Duke Social Support Index (DSSI)*, psychological stress scale, *Dickman Impulsivity Inventory (DII), State-Trait Anxiety Inventory (STAI), Center for Epidemiologic Studies Depression Scale (CES-D)*, and *Beck Hopelessness Scale (BHS)*. The mean suicidal intent score was 14.23 ± 6.22. Linear regression analysis revealed that nuptiality, living area, impulsivity, hopelessness, depression, psychological strain, and social support impacted suicidal intent. The study concluded that unmarried individuals living in cities and struggling with high levels of psychological tension, despair, and depression, merit appropriate interventions to reduce the incidence of intentional APP and suicide.

Lastly, Rajkumar's brief report provides the findings of a pilot study looking at the association between national suicide rates in males and females and several socioeconomic indices (subjective wellbeing, sustainable development, type of political regime, economic and gender inequality, and social capital). Findings identified the *Happy Planet Index* (a composite measure of subjective wellbeing and sustainable development) was negatively associated with suicide rates independent of gender. Alternatively, economic inequality was associated with suicide in male while social capital was associated with suicide in female. These results emphasize the importance of integrating sociocultural factors into national suicide prevention programs.

To conclude, the papers compiled in this Research Topic provide valuable insights into the measurement of suicidal behavior and provide insights for the development of effective prevention strategies. Moreover, investigating potential biomarkers provides new opportunities to identify individuals who may be at risk of experiencing suicidal thoughts. It is crucial for future research to further explore the complex aspects of suicidal behavior and prioritize the implementation of evidence-based interventions in order to alleviate the global burden of suicide.

## Author contributions

All authors listed have made a substantial, direct, and intellectual contribution to the work and approved it for publication.
